# Laminoplasty Techniques for the Treatment of Multilevel Cervical Stenosis

**DOI:** 10.1155/2012/307916

**Published:** 2012-03-06

**Authors:** Lance K. Mitsunaga, Eric O. Klineberg, Munish C. Gupta

**Affiliations:** Department of Orthopaedic Surgery, University of California Davis Medical Center, 4860 Y Street, Suite 1700, Sacramento, CA 95817, USA

## Abstract

Laminoplasty is one surgical option for cervical spondylotic myelopathy. It was developed to avoid the significant risk of complications associated with alternative surgical options such as anterior decompression and fusion and laminectomy with or without posterior fusion. Various laminoplasty techniques have been described. All of these variations are designed to reposition the laminae and expand the spinal canal while retaining the dorsal elements to protect the dura from scar formation and to preserve postoperative cervical stability and alignment. With the right surgical indications, reliable results can be expected with laminoplasty in treating patients with multilevel cervical myelopathy.

## 1. Introduction

While multilevel cervical stenosis may occur for a variety of reasons, it is usually due to cervical spondylosis or ossification of the posterior longitudinal ligament (OPLL). Options for decompression of the canal include either anterior or posterior approaches. For multilevel disease, most surgeons prefer posterior decompression. Posterior decompression has the advantage of addressing multiple levels with one incision. However, this approach is hindered by the late complication of kyphosis with decompression alone or the loss of motion and adjacent segment degeneration if posterior decompression is performed in conjunction with fusion [1, 2]. Laminoplasty is a technique that indirectly decompresses the spinal cord and preserves neck motion by avoiding fusion. This is accomplished by hinging the laminae open on one or both sides to allow the spinal cord to migrate posteriorly away from anterior compressive structures. Laminoplasty was initially described by the Japanese in the early 1970s to treat ossification of posterior longitudinal ligament [3]. By leaving the dorsal structures in situ, laminoplasty was developed to avoid the problems associated with laminectomy, such as kyphosis, instability, and delayed neurologic problems due to scar invasion. Laminoplasty has become increasingly popular in North America as experience with laminoplasty techniques has grown and its application has expanded to treat other causes of multilevel cervical stenosis besides OPLL, such as cervical spondylotic myelopathy (CSM). The goals of this chapter are to discuss the advantages and disadvantages of laminoplasty, key technical points regarding different laminoplasty techniques, along with the complications and outcomes of laminoplasty.

## 2. Advantages of Laminoplasty

Laminoplasty allows the spinal cord and the neuroforamen to be decompressed without directly removing anterior pathology. By preserving the dorsal elements of the spine, laminoplasty preserves spine stability and alignment and decreases the risk of postlaminectomy kyphosis and instability [1, 4–23]. Additionally, since fusion is not required, complications such as fixation failure, pseudarthrosis, loss of motion, and adjacent segment degeneration do not occur. This may allow earlier mobilization and rehabilitation compared to other surgical options. In addition, laminoplasty can avoid graft-related complications such as graft extrusion, settling, collapse, dislodgement, and fracture. The financial costs associated with laminoplasty are potentially minimized, as well, without the need for lateral mass screws and rods used during fusion. With laminectomy, epidural scar formation can form between the dura and muscle leading to postoperative pain and neurologic compression [24–26]. However, with laminoplasty, the lamina is preserved and it protects the dura from this “postlaminectomy membrane.” Preserving the lamina also makes revision procedures requiring posterior approaches safer. Finally, laminoplasty has the advantage of avoiding the potential morbidity that can accompany anterior approaches, such as dysphagia, recurrent laryngeal nerve injury, dysphonia, and injury to the esophagus or carotid sheath contents.

## 3. Disadvantages of Laminoplasty

Laminoplasty does not address neck pain, if that is a component of a patient's symptomatology. Laminoplasty may even cause worse neck pain than anterior procedures, especially in the early postoperative period, due to the extensive muscle stripping that accompanies this procedure. This has been shown to be especially true if the dissection and laminoplasty is carried down to C7 [27, 28]. Although laminoplasty does not require fusion, range of motion is still reduced following laminoplasty [4, 8, 18, 20, 29–37]. Once the laminoplasty hinge has been opened, it requires some stabilization to maintain the expanded position during healing. This fixation is associated with some cost, bone graft may be required, and there is the potential for nonunion or failure. In addition, the longitudinal incision associated with laminoplasty may be less cosmetic than anterior incisions.

## 4. Indications

Cervical laminoplasty is indicated for cases of cervical myelopathy or myeloradiculopathy due to central stenosis extending more than three intervertebral disc spaces. This can be due to multilevel degenerative cervical spondylosis, OPLL, multilevel disc herniations, certain spinal cord tumors, neuromuscular disorders, acute traumatic central cord syndrome, or developmental cervical stenosis.

## 5. Contraindications

Kyphotic deformity is a contraindication for laminoplasty. In the kyphotic spine, laminoplasty does not address the cord compression anteriorly and leads to decreased canal expansion and dorsal migration of the cord [38]. In addition, laminoplasty can contribute to spine instability and worsen the kyphosis in these cases. Ideal laminoplasty patients have lordotic cervical spine alignment and no instability on dynamic radiographs. Laminoplasty can be done in neutral spines (generally defined as less than 4 degrees of either kyphotic or lordotic angulation) but lordotic alignment is preferable since multiple studies have documented loss of lordosis after laminoplasty [12, 20, 29, 30, 39–51]. In patients with radiographic evidence of instability, laminoplasty alone may worsen the instability and should be accompanied by fusion. The ideal laminoplasty patient also has minimal complaints of neck pain. Laminoplasty may not address neck pain and, in fact, may even worsen neck pain. However, the presence of mild neck pain is not a contraindication to laminoplasty, provided the patient accepts the risk of postoperative neck pain. Stenosis at one or two levels is not an indication for laminoplasty since the short length of decompression achieved in these cases does not yield the same amount of spinal cord migration away from anterior structures. Post-laminoplasty instability may also be an issue in patients with rheumatoid arthritis, which is a relative contraindication for laminoplasty [52].

## 6. Preoperative Considerations

As always, a thorough history and physical exam, especially a thorough neurologic exam, are imperative prior to performing a laminoplasty. Radicular symptoms may suggest the need for a foraminotomy, while significant neck pain may indicate the patient for posterior fusion in combination with laminoplasty. AP/lateral views of the cervical spine and CT myelogram or MRI of the cervical spine is also critical. These imaging modalities can be used to correlate with clinical findings and to assist with surgical planning. They are also important if cervical foraminotomy is needed as they can be used to help assess the level(s) and location of nerve root compression. Upright plain films are used to determine preoperative kyphosis and one's ability to perform posterior surgery. Flexion-extension films should be evaluated for any evidence of instability that may need to be addressed with a posterior fusion.

## 7. Patient Positioning

We prefer to use the 180-degree operating room setup and a Mayfield tong attachment with gelfoam bolster pads on a standard table. The Mayfield tongs allow control and stability of the cervical spine during positioning. Other alternative operating room table and positioning options include a Jackson frame with a Mayfield tong attachment or a Stryker or Jackson frame with Gardner tong traction.

Intubation must be achieved cautiously. In cases involving severe myelopathy, awake fiberoptic intubation is often recommended to minimize the risk of spinal cord injury with neck extension and to allow monitoring of neurologic status after intubation. Neuromonitoring electrodes are placed and baseline somatosensory and transcranial motor evoked potentials are recorded prior to commencement of the case. Mayfield tong retractors are placed and the patient is placed prone.

**Figure 1:**
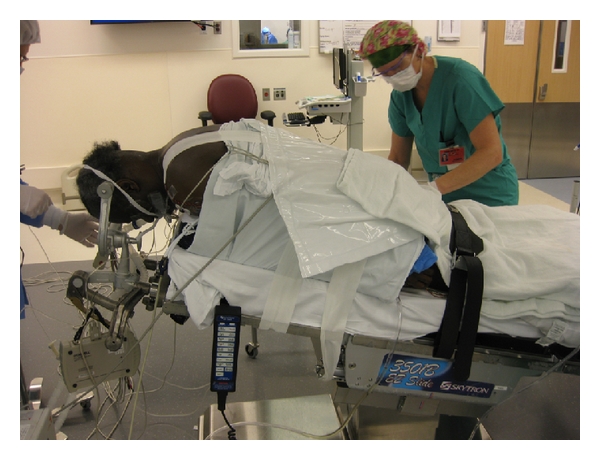
Photo demonstrating proper patient positioning. Patient is prone and the head is secured by Mayfield tongs without traction. The table is placed in the reverse Trendelenburg position. Shoulders are taped down and the neck is in slight flexion.

The table should be placed in reverse Trendelenburg position at about 20–30 degrees to help with venous drainage and visualization. The shoulders are taped down to provide more complete radiographic visualization of the lower cervical spine. The head should be in maximum capital flexion and the cervical spine in a neutral position to maximize interlaminar and interspinous space and to open the facet joints during foraminotomy. The exception to this is if posterior fusion is planned, in which case slight extension of the neck during positioning or prior to fusion is necessary. The preferred patient positioning setup is shown in Figure 1. After patient positioning, SSEP and MEP signals are again checked to see if there is any change from baseline that might require repositioning. The posterior cervical spine is then prepped and draped in the usual sterile fashion, including the occipital protuberance rostrally to the T3 spinous process caudally.

## 8. Laminoplasty Techniques

**Figure 2:**
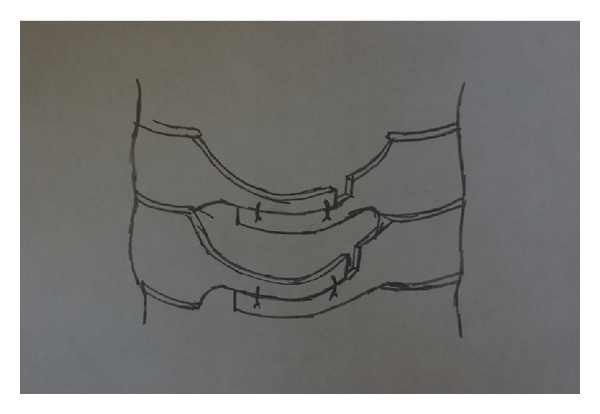
In the original “Z-plasty,” after troughs are drilled at the junction between the lamina and lateral mass, the laminae are thinned, a “Z” is cut in the laminae with a drill, and, as shown here, the laminae are spread apart and held with wire or suture to maintain the expanded canal position.

Oyama et al. first described cervical laminoplasty in Japanese in 1973 as a treatment for OPLL [3]. In this initial expansive laminoplasty procedure, the “Z-plasty” of the cervical spine, the spinous processes are removed, the lamina is thinned to the lamina-facet junction, and a Z-shaped cut is made between the laminae which are opened and fixed with suture or wire (Figure 2). Since its initial description by Oyama, laminoplasty techniques have been constantly refined. Most of these changes relate to how the cuts in the lamina or spinous process are made and how the laminae are secured in an open position—with wires or heavy sutures, bone anchors or bone blocks, hydroxyapatite blocks, miniplates, local spinous process autograft, and combinations thereof. All variations in laminoplasty techniques maintain the common theme, however, of repositioning the laminae, expanding the canal, and preserving the dorsal elements to maintain stability. In general, none of these technical variations have proven to be any safer or efficacious than the other. There are generally three categories of laminoplasty techniques: the “open door” laminoplasty, the “double door” laminoplasty, and the various muscle-sparing laminoplasty techniques. We will describe these techniques in detail in the following sections.

## 9. Expansive Open-Door Laminoplasty (Also Known as the Hirabayashi, Open-Hinged, or Single-Door Technique)

Hirabayashi et al. simplified the Z-plasty described by Oyama in the early 1980s with his unilateral expansive open-door laminoplasty [4, 34]. In this technique, a hinge is created on one side of the lamina-spinous process-ligamentum flavum complex. This allows the roof of the canal to be opened on the contralateral side leading to an expansion of the spinal canal.

**Figure 3:**
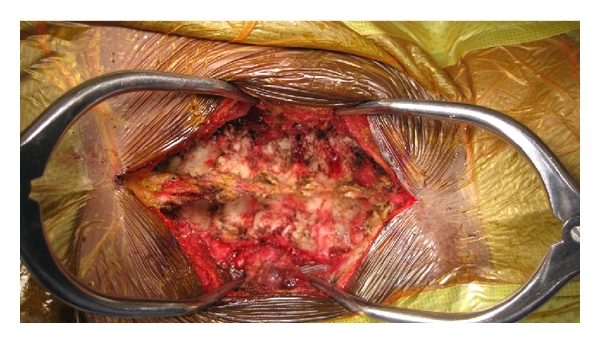
Intraoperative photo showing laminoplasty exposure. Following midline dissection along the avascular subperiosteal plane, paraspinal muscles are retracted laterally and the spinous processes, lamina, and medial aspect of the facets should be completed denuded of soft tissue.

Most commonly, laminoplasty is performed from C3 to C7 and all these levels need to be exposed. This starts with a midline longitudinal posterior incision from the occipital protuberance down to the T1 spinous process. With electrocautery, dissection proceeds through the midline fascia and ligamentum nuchae and the spinous processes from C2 to T1 are exposed. Levels can be identified by palpation and visualization of the prominent, bifid C2 spinous process. Preserve the supraspinous and interspinous ligaments at the proximal and distal extents of the exposure during this approach but Bovie electrocautery can be used to incise these ligaments in the midline at the planned laminoplasty and foraminotomy levels. Also preserve the muscular attachments to C2 as much as possible to minimize risk of postoperative kyphotic deformity between C2 and C3. This is facilitated by first exposing the C7 lamina, retracting the paravertebral muscles at this level, and continuing this dissection rostrally to the upper part of the C3 lamina. Complete this midline, subperiosteal dissection of the paravertebral muscles for the C3 to C7 spinous processes and retract these muscles laterally off the spinous processes, laminae, and medial aspect of the facets. Staying in the natural, avascular, subperiosteal plane prevents damage to the paraspinal muscles and minimizes blood loss.  Figure 3 shows the exposure required to perform a laminoplasty. This dissection needs to be extended as far as the lateral masses but the facet capsules must be preserved unless fusion is being performed. Hemostasis at all times can be achieved with monopolar or bipolar electrocautery.

Again, the C2 extensor muscular attachments do not need to be released. If, however, a decompression is necessary at the C2 level and undercutting of the lamina with a burr is not adequate, a laminoplasty can be done at C2 as well. In this case, the C2 extensor muscles can be released or taken off with a thin osseous sleeve and subsequently sutured back down to the C2 dorsal structures. If a foraminotomy is planned at C2-C3, the extensors on the inferior half of C2 are released so the facets can be visualized adequately. At this point, once the soft tissues have been reflected off the spinous processes and laminae, retractors can be placed on the sides of the wound and, if necessary, rostrally and caudally, to facilitate visualization. The microscope may also be extremely helpful for visualization, especially during foraminotomy, decompression, and creation of the laminar osteotomies.

**Figure 4:**
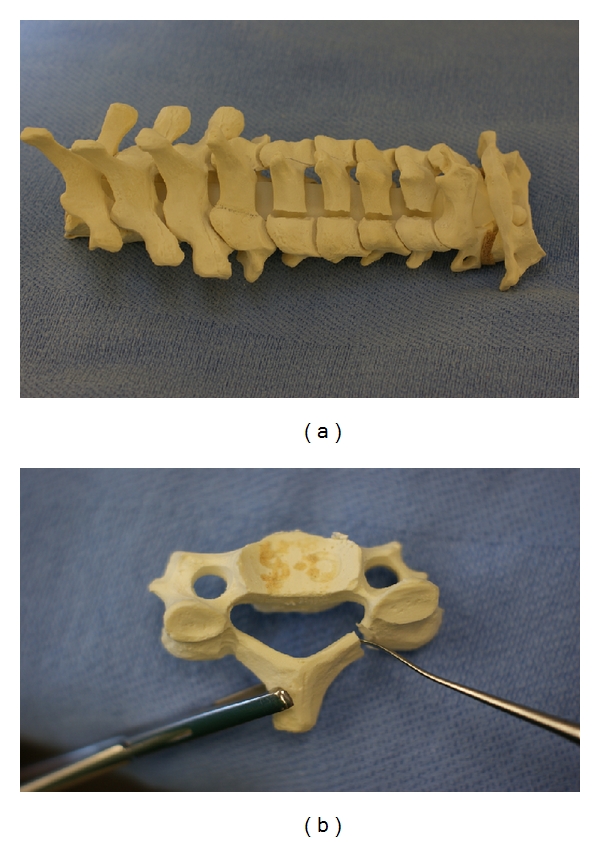
Sawbones model of an open-door laminoplasty. A trough has been drilled in the lamina at the lamina-lateral mass junction with a burr until there is a thin remnant of lamina left. This cut has been completed with a Kerrison punch (a). After the complete trough on the open side, a second trough is drilled on the hinge side. Expand the canal and the opening on the open side with a curette on the open side between the laminae and lateral mass while rotating the lamina towards the hinged side with a Kocher, as shown in (b).

In the expansive open-door laminoplasty, the opening side of the lamina should be cut before the hinged side to minimize blood loss. The opening side is usually placed on the side with worse radicular symptoms or more stenosis because, on this side, it is technically easier to perform a foraminotomy to decompress the neuroforamen. If it is necessary to perform a foraminotomy on the hinged side, this should be done first to prevent detachment of the lamina and because this is technically more demanding. Using controlled, side-to-side brushing motions with a 2 or 3 mm cutting burr or high-speed microdrill, a trough is made at the junction of the lamina and lateral mass from C3 to C7 by decorticating the posterior aspect of the lamina (see Figure 4(a)). Extra caution should be used at the superior aspect of the lamina, where there is no ligamentum flavum to protect the dural sac. The troughs, created rostral to caudal from one level above to one level below the stenotic levels, should be perpendicular to the lamina. The facets should not be violated. Following thinning of the lamina down to a thin cortical layer, use a curette to free the ligamentum flavum off the inferior aspect of the C7 lamina. Complete the laminar cuts just medial to the pedicles with a 2 or 3 mm Kerrison punch by removing the thin rim of remnant lamina and associated ligamentum flavum from caudal to rostral. Epidural venous bleeding can be controlled with thrombin gel and bipolar electrocautery.

Next, for the “hinged” side of the laminoplasty, another trough is made at the junction of the lamina and lateral mass with minimal disruption of the facet capsule to prevent postoperative instability. While a 2 mm cutting burr or high-speed drill can be employed for this portion of the procedure, we find that a 6 mm diamond burr is useful to cut the outer cortex in the process of creating a greenstick fracture on the hinged side as it minimizes the risk of completely breaking through the inner cortex of the lamina. This large burr tip also helps create a slightly wider trough on this hinged side so the walls of the trough do not contact each other when the door is opened which would limit the amount of decompression. Again, the lamina is thinned with a burr by removing the outer cortex and approximately one half of the cancellous bone. Do not violate the inner cortex which will act as a hinge. To prevent excessive thinning of the lamina on the hinged side and complete dissociation of the entire lamina, it is helpful to periodically assess the amount of “give” in the spinous process when it is manipulated and to use the depth of the lamina on the opened side as a reference for how deep the trough should be on the hinged side. Additionally, a curette can be placed on the open side and pulled upwards. When motion in the lamina is seen with this maneuver, the trough on the hinged side is complete. The top and bottom laminae of the open side which will be included in the laminoplasty can be separated from adjacent levels with a Kerrison Rongeur to cut the lamina and attached ligamentum flavum. This creates three free borders for the “door” which can now hinge open.

Once the laminae are thinned sufficiently, the posterior elements are more flexible and the lamina can be opened very gradually and carefully by additional thinning of the hinged bone, pulling the spinous process toward the hinged side, and lifting the lamina off the spinal cord with a curet on the opening side. Opening the laminae as a single unit preserves the intraspinous ligaments and dorsal structures that help stabilize the spine. This can be accomplished by opening the lamina at each level gradually and to the same degree as the other. In this way, the gap on the open side between the lamina and facet is increased and a greenstick fracture is established along the trough on the hinged side. If this step is performed with too much force or speed, the inner cortex on the hinged side can fracture. In this case, laminectomy with fusion or stabilization of the hinged side is required. Additionally, if control of the lamina is lost and the door inadvertently snaps closed, this can injure the spinal cord. A Penfield dissector or curette can be helpful to expand the opening and the lamina can be rotated towards the hinged side with a Kocher (see Figure 4(b)). A Woodsen probe or elevator can be used to release any adhesions between the dura and ventral lamina on the opened side. Laminar opening is made easier with posterior elements that are more pliable, and this can be achieved with division of the supraspinous and interspinous ligaments at C2-C3 and C7-T1 and/or release of the ligamentum flavum on the opening side of the lamina. Once pulsatile flow in the dura is noted, which is usually at about 8–10 mm of opening, adequate canal expansion has been achieved. Hemostasis is achieved with gelfoam or surgicel and epidural bleeders are controlled with bipolar electrocautery. Bony bleeding is controlled with bone wax on a kittner or other local hemostatic agents.

In his initial description, Hirabayashi stabilized the laminae in an open position with sutures through the facet capsule and spinous processes on the hinged side. Titanium miniplates with or without allograft or autograft, allograft stabilized with CG clips, stainless steel wiring, facet cables with and without allograft, various suture techniques, ceramic implants, bone anchors placed through the lateral mass, and various structural wedge allografts and autografts are other modified techniques that have been described to keep the door propped in the open position [6, 46, 47, 53–58]. The senior authors prefer either a suture anchor or miniplate for initial stabilization.

**Figure 5:**
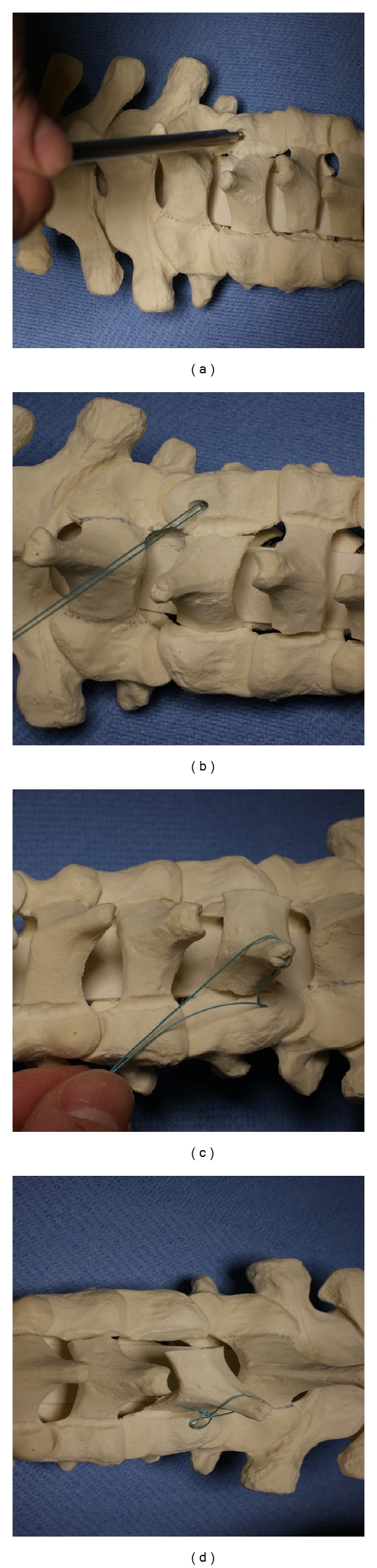
Once the door is opened, the laminae can be held in the open position with suture anchors. Suture anchors are placed into the lateral masses of the hinge as shown in (a) and (b). Next, the suture anchor is brought through a drill hole in the spinous process (c). The suture is tied to prevent closure of the laminoplasty (d).

 Suture anchors are a simple way to maintain the opening of the laminae. We find them to be a safe and time-efficient method that minimizes the risk of disrupting the facet joints and nerve roots. In addition, this technique does not require grafting or fixation of the lamina to the lateral mass. The suture anchors are placed into the lateral masses on the hinged side (Figures 5(a) and 5(b)). We make a hole in the base of the spinous process using a right-angle dental drill with a 2 mm burr tip. Once the laminae are hinged open, a Keith needle is used to bring the nonabsorbable suture from the suture anchor in the lateral mass through the drill hole in the spinous process (Figure 5(c)). Appropriate tension on the suture is achieved with a slip knot and then square knots are tied under tension to maintain the laminar opening (Figure 5(d)).

**Figure 6:**
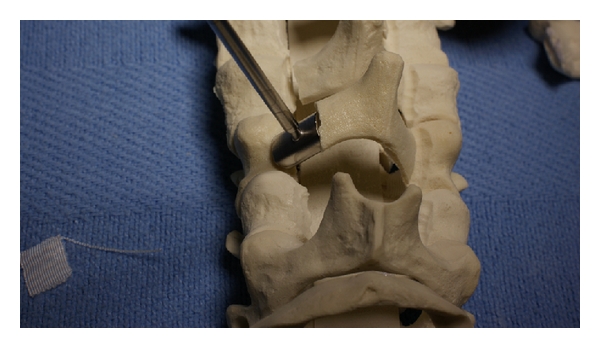
For titanium miniplate fixation, once the laminae are expanded, use a trial spacer to determine the appropriate allograft size.

**Figure 7:**
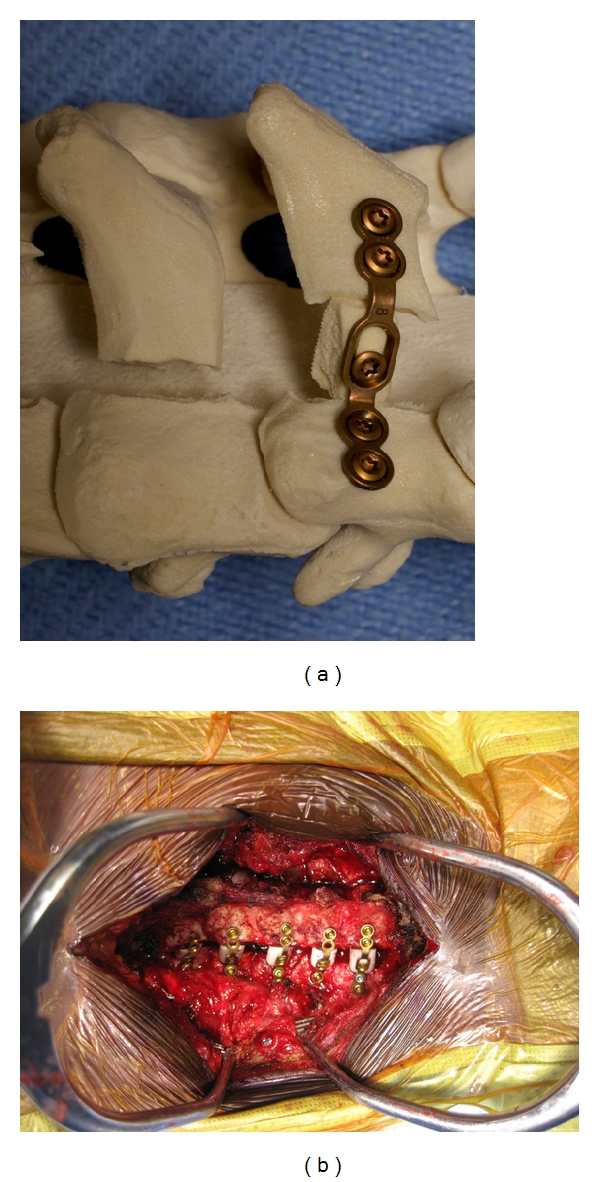
Sawbones model (a) and intraoperative photo (b) showing miniplate and allograft placed in the laminar opening. The miniplate has been fixed with 2 screws on both the laminar and lateral mass side.

Our other preferred fixation method involves using titanium miniplates and allograft spacers to hold the laminae open, as shown in Figure 7. Once the laminae are expanded, the appropriate allograft size is ascertained by inserting a trial spacer into the laminar opening (Figure 6). We then choose a double pre-bent miniplate of appropriate length which can be fixed to the allograft via a center screw hole. The miniplate-allograft construct is then placed in the laminar opening such that the cut laminar edges are wedged securely in the ends of the allograft that are prenotched. The graft should fit securely in the laminar gap. We then secure the miniplate with one or two self-tapping 2.0 mm cortex screws on both the laminar and lateral mass side.

We have found both suture anchors and miniplate fixation to be reasonable methods of maintaining the laminar opening, as shown in Figure 9.

## 10. Double-Door Laminoplasty (Also Known as French Door, Spinous Process-Splitting, Midline Opening, or T-Saw Laminoplasty)

In Hirabayashi's expansive open-door laminoplasty, the spinal cord is decompressed asymmetrically since the door opens on one side and hinges on the other. In contrast, the double-door laminoplasty, described by Kurokawa in 1982, expands the canal symmetrically as the opening is created in the midline [59]. This is accomplished by splitting the spinous processes in the midline with the left and right hemilaminae hinging on the lamina-spinous process-ligamentum flavum complex bilaterally (Figure 10(a)). In the double-door technique, the same positioning, draping, and midline posterior exposure as the open-door technique is performed. This exposure is carried out laterally to the middle of the lateral masses. Preserve the semispinalis muscle attachments to the C2 spinous process as much as possible. Troughs are drilled bilaterally with a high-speed drill or burr at the lamina-lateral mass junction from C3 to C7, just medial to the pedicle (Figures 10(b) and 10(c)). The inner cortex, as with the open-door technique, is only thinned. The spinous processes are then split down the middle. A drill or burr is used to thin the lamina and a Kerrison punch is used to open the lamina in the midline. The midline laminar splits can be opened with laminar spreaders (Figure 10(d)). The laminae are lifted off the spinal cord in the midline and held open like a French door. This allows the spinal cord to drift posteriorly in the enlarged canal.

One purported advantage of the double-door technique is that the decompression occurs directly posterior to the cord so there is less bleeding from the lateral epidural veins that often accompanies the open-door technique. Adhesions between the dura and ventral side of the lamina are freed. The laminar opening can be fixed with suture passing through the facet capsules and lamina. In the initial description of the double-door procedure, the canal was left open. However, several techniques have been proposed to span the space between the gapped lamina and to protect the spinal cord. These include the use of ceramic/hydroxyapatite spacers, iliac crest bone graft, rib autograft (Figure 10(e)), or, as described by Kurokawa, resected spinous process autograft fixed between the lamina with wires [59–61].

In the Tomita modification of the double-door laminoplasty (also known as the “T-saw laminoplasty”), the spinous processes and laminae are split with a Gigli-like wire-saw [29, 62] (Figure 11). After the ligamentum flavum is resected down the midline above and below the levels to be decompressed, a polyethylene sleeve that encompasses a T-saw is passed superiorly along the epidural space. Grab hold of the sleeve tip as it comes into view in the C2-C3 interspace. Advance the saw through the sleeve and remove the sleeve over the saw. After verifying adequate cervical lordotic alignment to minimize the risk of cord injury, a reciprocating sawing motion is used to saw through the midline of the laminar arch. Irrigate periodically to minimize thermal damage. Greenstick fractures are then created by using a high-speed burr to create bilateral troughs at the medial one-third of the lateral masses. The split laminae are then opened as with the double-door technique. Carefully free up any epidural adhesions. Foraminotomies can be done prior to opening the hinges. The opened canal can be stabilized with various grafts, including rib or fibula allograft spacers, autologous spinous process, or iliac crest [29, 63].

The primary disadvantage of the double-door technique is that it can be technically challenging. This technique also potentially puts the spinal cord more at risk than the open-door technique as the dura is just deep to the spinous process that is split with a burr or saw. Foraminotomy is also technically demanding and may cause disruption of the hinge.

## 11. Muscle-Sparing Laminoplasty Techniques

Many problems associated with laminoplasty such as axial neck pain, postoperative kyphosis, and segmental instability are thought to be related to neck muscle disruption [12, 33, 64–66]. Various techniques have been described to minimize disruption of muscular and ligamentous attachments to the lamina and spinous processes.

Shiraishi described a technique designed to minimize damage to the deep extensor muscles of the cervical spine, and, in particular, the attachments of the semispinalis cervicis (SSC) and multifidus muscles to the spinous processes [65] (Figure 12). An operating microscope is recommended for this minimally invasive exposure. A longitudinal midline incision is made overlying the spinous processes of the planned laminoplasty levels. For instance, for a C4 and C5 laminoplasty, the incision would overlie the C4 and C5 spinous processes. The nuchal ligament is incised in line with this incision. Identify the interval between the tips of the C4-C5 spinous processes, which separates the insertions of the interspinalis muscles, SSC, and multifidus muscles to the C4 spinous process from the left and right side. This interval is opened with a nerve retractor such that the muscles attached to the C4 spinous process on the left are retracted to the left and vice versa for those muscles attaching on the right side of the C4 spinous process. The ligamentum flavum and superior half of the C5 lamina should be visible. Sever the interspinalis muscles where they attach to the C5 spinous process. The attachments of the SSC and multifidus muscles on the C5 spinous process are pulled distally so that the attachments of the rotator muscles inserting on the inferior half of the C5 lamina can be seen and dissected off the C5 lamina. By retracting the muscles attached to the C5 spinous process even more distally and those to the C4 spinous process laterally, the C5 lamina, the superior border of the C6 lamina, and the C5-C6 intervertebral joint are exposed without taking down any of the SSC or multifidus attachments to the C4, C5, or C6 spinous processes. Following this exposure that preserves the attachments of the SSC and multifidus to the spinous processes, a double-door laminoplasty can be performed similar to its application with the standard midline posterior approach.

Others, however, have suggested that it is not disruption of the extensor muscles that causes axial pain, but disruption of the musculoligamentous structures attached to the C7 spinous process, such as the trapezius, rhomboid minor, and the nuchal ligament [27, 28, 67]. This has led to the recommendation that C7 should be excluded, if possible, from the laminoplasty procedure. This may minimize postoperative neck pain by preserving the C7 spinous process as a fulcrum for neck muscles and it maintains the stabilizing role the C7 lamina plays in the cervical spine [27, 28, 67]. Alternatively, if the epidural space at the ventral aspect of the C7 lamina is tight upon probing, a laminotomy at the superior half of C7 can be done [67].

Others, still, have focused not on disruption of the C7 musculoligamentous attachments as a major source of axial neck pain, loss of extensor power, and loss of cervical alignment, but, instead, disruption of the extensor musculature and, in particular, the semispinalis cervicis muscle attachments on C2 [21, 32, 33, 68–75]. A traditional C3-C7 laminoplasty usually requires disrupting and reattaching the extensor muscle insertions onto C2. Takeuchi, however, proposed a C3 laminectomy with a C4-C7 laminoplasty to preserve these muscles and minimize axial neck pain [69]. In another muscle-sparing modification, some recommend a C3 laminectomy, a laminoplasty from C4 to C6, and an undercutting of the inferior two-thirds of the lamina of C2 and superior half of the lamina of C7. The goal of this technique is to achieve an adequate decompression at C3-C7 but to minimize the incision, the potential for instability, and the amount of bony work and muscle disruption. In another muscle-sparing modification aimed at preventing post-laminoplasty cervical malalignment, Shiraishi and Yato proposed a variation of the double-door laminoplasty procedure that expands the C2 spinal canal while preserving all the muscular attachments to each half of the split C2 spinous process [70].

## 12. Foraminotomy

Performing a foraminotomy in association with laminoplasty is indicated in cases of significant radiculopathy or if there is radiographic evidence of neuroforaminal stenosis regardless of symptoms. This is done both to prevent the development of nerve root compression postoperatively and, possibly, to minimize postop neck pain due to nerve root stretch and compression. Some also recommend performing a foraminotomy based on abnormal neuromonitoring signals, especially those involving the C5 nerve root, which is particularly vulnerable to traction injury. Others, still, routinely perform bilateral C4-C5 foraminotomies to minimize the risk of a C5 root palsy.

The use of an operating microscope can be helpful for visualization purposes during the foraminotomy. The opening side of the laminoplasty should be placed on the side of the radiculopathy since this is the side that neuroforaminal decompression is easiest. Foraminotomies are usually done once the lamina is raised and the ligamentum flavum is removed. Foraminotomy is done by deroofing the foramen—that is, removing the superior articular facet of the caudal segment that covers the foramen, which is generally the medial third of the facet. This posterior decompression allows the nerve root to migrate posteriorly, away from the uncovertebral osteophytes. If a foraminotomy is to be performed on the hinge side, it should be completed before the laminoplasty to prevent complete disruption of the lamina.

A high-speed burr is used to remove about 50%, medial to lateral, of the inferior articular facet which overlies the superior articular facet dorsally. This exposes the articular surface of the superior articular facet. Thin the facet with a combination of cutting and diamond burrs and then a 1 mm Kerrison punch is used to remove the roof of the foramen, thus exposing the exiting nerve root. Adequate decompression requires resection of the superior articular facet overlying the inferior pedicle but not lateral to it, as this may lead to instability. Verify adequate decompression by probing the foraminal opening with a nerve hook. The lateral wall of the superior and inferior pedicles should be easily palpable. Hemostasis is facilitated with gelfoam and thrombin.

## 13. Fusion

Fusion is indicated with laminoplasty for patients with severe axial neck pain, evidence of instability, or bilateral radicular symptoms in addition to myelopathy. If fusion is planned, the exposure needs to be carried out laterally to the lateral aspect of the facet joints. Additionally, excision of facet capsules from C3 to C7 should be performed during the exposure. After the laminoplasty is complete, place lateral mass screws at C3 to C7. The start point is 1 mm medial to the center of the lateral mass with a 15-degree rostral and 30-degree lateral trajectory [76]. Some prefer a pedicle screw at C7 for additional stability. After lateral mass decortication, allograft or cancellous iliac crest autograft is packed into the decorticated fusion bed.

## 14. Wound Closure

**Figure 8:**
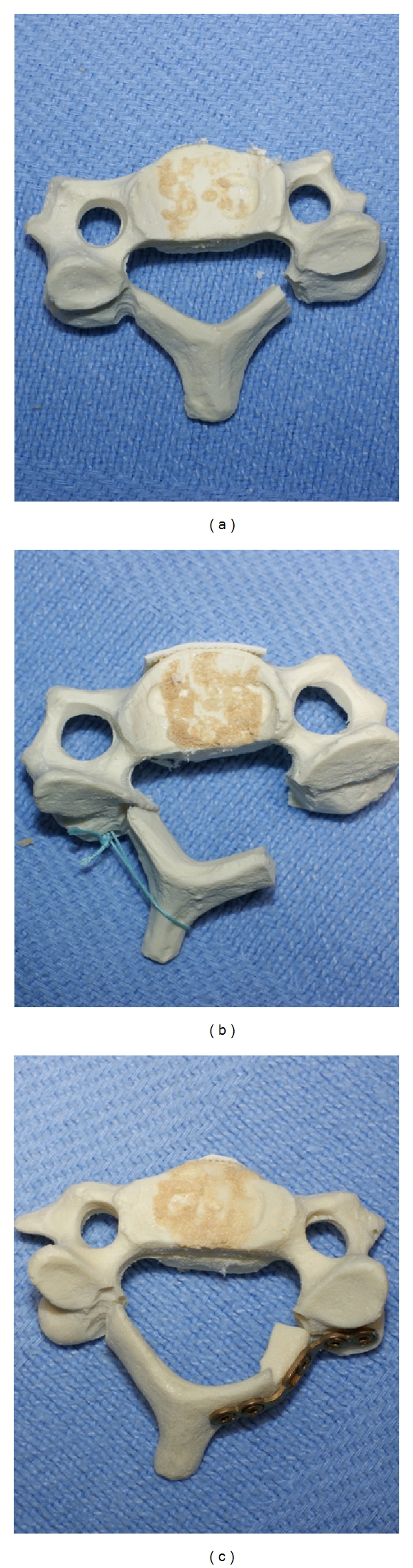
Sawbones model showing an increase in A-P diameter of the canal between the preexpanded status (a), after suture anchor laminoplasty (b), and after laminoplasty with miniplate fixation (c).

**Figure 9:**
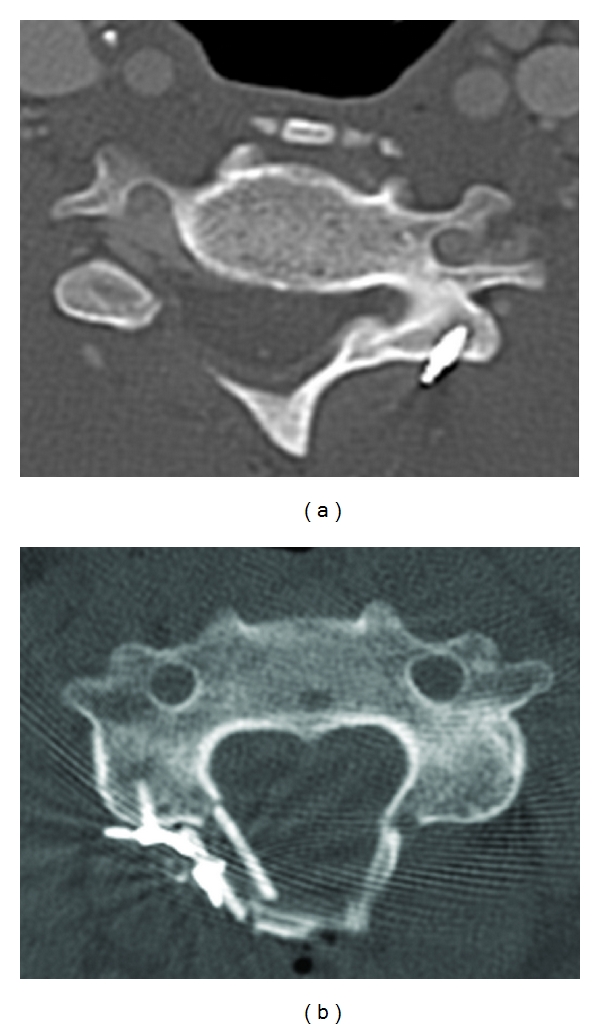
Axial CT scans of patients following expansive open-door laminoplasty. The laminar opening has been maintained with suture anchors (a) and miniplate fixation (b).

**Figure 10:**
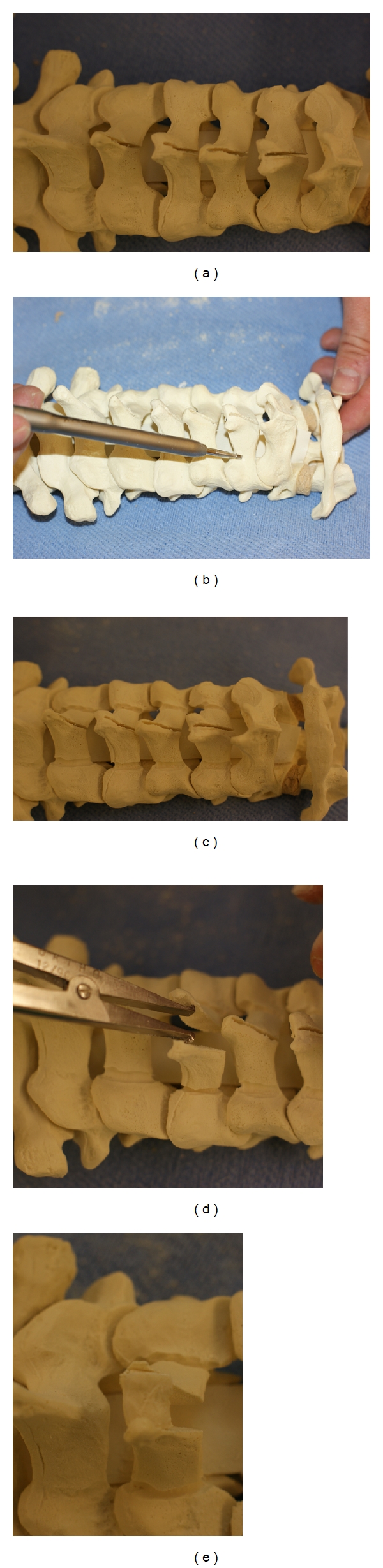
In the double-door laminoplasty technique, a high-speed burr is used to split the spinous processes in the midline (a). The burr is then used to create troughs bilaterally at the junction of the lamina and lateral mass, as shown in (b) and (c). The hemilaminae are separated at the midline with a laminar spreader and elevated (d). The expanded canal is then held in the open position with either bone graft or ceramic/hydroxyapatite spacers (e).

**Figure 11:**
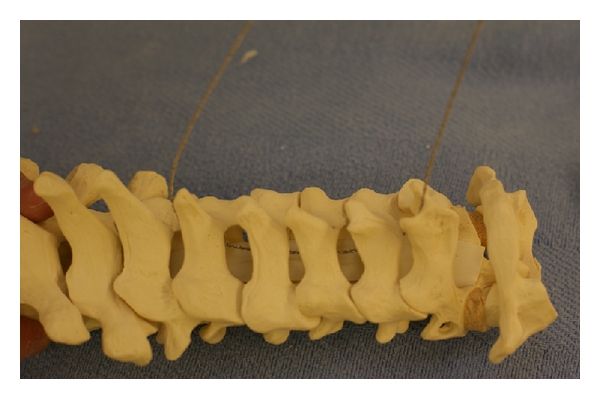
Instead of using a burr for the double-door laminoplasty technique, with the Tomita modification of the double-door laminoplasty, the spinous processes are split in the midline with a Gigli-like T-saw.

**Figure 12:**
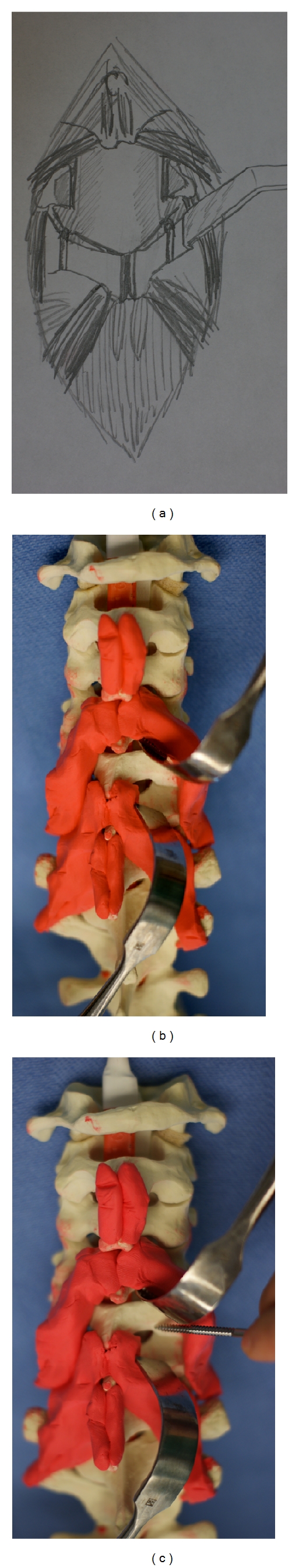
Illustration (a) and sawbones model (b) showing how a muscle-sparing exposure can be applied to a double-door laminoplasty. The deep extensor muscles attaching to the C4 spinous process are pulled laterally and those attaching to the C5 spinous process are pulled distally. The double-door laminoplasty can then be done as with the standard posterior midline exposure with sagittal splitting of the spinous processes and the creation of bilateral laminar troughs which serve as hinges on which the double-door can open, as shown in (c).

After copious irrigation of the wound with normal saline, retractors are removed, hemostasis is achieved, and a deep drain is placed. A standard layered closure is then performed. We close the fascia covering the paravertebral musculature with a No. 0 vicryl suture in a Figure 8 fashion, followed by a No. 0 PDS running stitch. The subcutaneous layer is closed with No. 2-0 vicryl in an interrupted buried fashion. We close the skin with either a No. 3-0 monocryl suture using a running subcuticular technique or a No. 3-0 nylon baseball stitch.

## 15. Postoperative Management

The head of the bed should be kept at greater than 45 degrees for the first couple days after surgery to minimize venous bleeding. Patients are immobilized in a rigid cervical collar, such as an Aspen collar, for 3-4 weeks. Mobilization with physical therapy starts on postoperative day one, including bed transfers and ambulation. We obtain AP and lateral plain films of the cervical spine prior to patient discharge. Patients typically are discharged 24 to 48 hours after surgery and return for their first follow-up visit 3-4 weeks later, at which point we obtain repeat AP and lateral cervical spine films. We encourage patients to return to their day-to-day activities as soon as possible. At 3-4 weeks, patients can discontinue the use of their collar and initiate isometric neck exercises.

## 16. Complications

### 16.1. Wound Complications

Some argue that wound complications, such as infection or dehiscence, is a greater risk with laminoplasty compared to laminectomy as the lamina are rotated and held open [77]. This is an inherent complication for all posterior cervical approaches due to the strong muscular attachments. We have found the rate of infection and dehiscence to be extremely low in our laminoplasty patients. Avoidance of soft tissue complications is facilitated by paying meticulous attention to soft tissue handling, copious irrigation, thorough hemostasis, excision of necrotic soft tissue prior to closure, a watertight closure, subfascial drain placement, and perioperative antibiotics.

### 16.2. Neurologic Complications

One of the advantages of laminoplasty, compared to laminectomy, is the theoretical decreased risk of neurologic complications as laminoplasty does not involve placing any instruments between the lamina and dural sac. Neurologic deterioration is, however, still a potential risk with laminoplasty. This may be due to hematoma, inadequate decompression, traumatic surgical technique, restenosis or persistent stenosis due to inadequate raising of the lamina, fracture of the hinged lamina, or closure or dislodgment of the laminar opening [46, 51]. Laminar closure can be related to inadequate stabilization of the opening or hardware issues, such as broken miniplates. The incidence of canal restenosis is challenging to ascertain as CT and MRI scans are not routinely obtained and, when they are, long-term radiographic assessment of space available for the cord is rarely reported.

As with laminectomy, nerve roots can be mechanically injured during laminoplasty procedures, particularly during decompression with a drill or punch. Isolated nerve root injuries are a particular concern with laminoplasty, however, and they occur around five to 11% of the time [4, 20, 78, 79]. This complication presents primarily with motor weakness. Sensory deficits are a less common presentation. C5 is the most common nerve root affected. Although C5 palsies usually present 1–3 days after surgery with deltoid weakness and shoulder pain, the presentation can occur as late as 20 days postop [80]. It is not clear what causes the C5 nerve root palsy but some postulate it is related to a traction injury to the nerve root. Not only are the C5 roots shorter and less forgiving to traction injuries, C5 is also at the apex of the lordotic cervical curve and, in general, it is at the center of the laminoplasty [20, 37, 79–81]. Thus, the cord drifts posteriorly at C5 moreso than at other levels, which preferentially stretches the C5 nerve root. Other potential mechanisms of injury to the C5 nerve root with laminoplasty include intraoperative trauma to the C5 nerve root, dislodging of the lamina on the hinge side, preoperative neuroforaminal stenosis not adequately addressed intraoperatively, and preexisting spinal cord pathology [20, 78, 81–83]. Some recommend intraoperative transcranial motor evoked potential and spontaneous EMG monitoring to prevent C5 root injuries by performing a C5 foraminotomy if there are any abnormal signals to indicate the need to do so.

Treatment of nerve root palsies involves physical therapy and nonsteroidal anti-inflammatory medication. In general, complete or near complete recovery from the C5 palsy occurs spontaneously within one year but it can take up to six years to recover [37]. Some have recommended prophylactic foraminotomy and facetectomy to prevent a C5 palsy but this recommendation has not been borne out in the literature.

### 16.3. Axial Neck Pain

The true incidence of axial neck pain or stiffness following laminoplasty is variable in the literature. Yoshida et al., in their series, found that laminoplasty did not improve or cause neck or shoulder pain [84]. Hosono et al., however, demonstrated that axial neck pain was present in 60% of patients following laminoplasty in the postop period, a significantly higher incidence rate than that of his anterior fusion patients [66, 84]. This variation in the literature with regards to the true incidence of post-laminoplasty axial neck pain is also evidenced by Sani's meta-analysis of outcomes in 71 laminoplasty series including more than 2000 patients: he found that postoperative axial neck pain occurred in anywhere from 6 to 60% of patients and did not depend on the type of laminoplasty performed [50]. Postoperative neck pain is thought to be related to dissection around the facets and soft-tissue retraction, necrosis, and scarring [66, 77]. The neck pain begins in the early postop period and usually goes away within a year. Preventing postoperative neck pain and stiffness is the basis for recommending early neck range of motion. Nonsteroidal anti-inflammatory medications and physical therapy can be of benefit, although this has not been studied in the literature.

### 16.4. Loss of Cervical Motion

Although one advantage of laminoplasty is that it allows for decompression without fusion, studies have reported a decrease in cervical range of motion after laminoplasty. This loss of motion is in the range of 17–75% but, usually, a global loss of cervical motion of approximately 50% is seen [4, 29, 33–37, 41, 46, 48, 49, 53, 55, 85–87]. There is some controversy over the clinical significance of this loss of cervical motion. Some argue that range of motion after laminoplasty is crucial to addressing mechanical stress and avoiding adjacent segment degeneration and axial neck pain [47]. On the other hand, some propose that post-laminoplasty stiffness contributes to resolution of OPLL, protects the spinal cord by limiting dynamic motion, and maximizes the potential for neurologic recovery [41, 51, 88].

### 16.5. Loss of Cervical Alignment

No laminoplasty technique can prevent the development of some kyphosis postoperatively. The range of worsening cervical alignment in the literature varies from 22 to 53%, a complication that is not avoided with fusion [12, 20, 29, 30, 40–51, 88]. There is a paucity of literature on the correlation between kyphotic deformity and clinical or neurologic outcomes and even some data suggesting there is no such correlation [36, 48, 51, 88]. Augmenting a laminoplasty with modern instrumentation, however, has been shown to help preserve lordosis [6].

## 17. Clinical Outcomes

Multiple studies have shown that patients with cervical myelopathy due to cervical spondylosis or OPLL do reliably benefit from neurologic improvement following laminoplasty. Most studies report outcomes using the Japanese Orthopedic Association (JOA) scoring system, documenting mean preop and postop scores and rate of recovery. Recovery rates following laminoplasty of at least 50–70% are consistently reported in the literature, though recovery rates as high as 90% have been reported [8, 18, 20, 29, 36, 37, 44, 46, 47, 78, 86, 89–92].

Multiple authors have verified the reliable outcomes of laminoplasty in the short to midterm, but there only a few series that have been able to show that improvement in neurologic status following laminoplasty is maintained in the long-term. Kawaguchi et al. reviewed long term outcomes (greater than 10 years) in 133 patients with cervical myelopathy treated with laminoplasty [43]. The average preoperative JOA score was 9.1 points, and, postoperatively, it improved to 13.7 within one year. Although he did note some cases of neurologic decline, postoperative radiculopathy, kyphotic deformity, and loss of motion, JOA scores and recovery rates were maintained at 13.4 points and 55% at last followup, respectively. Seichi et al. performed a long-term retrospective study looking at the results of double-door laminoplasty in 35 patients with OPLL and 25 patients with CSM, including 5 patients with athetoid cerebral palsy [36]. Average followup was about 13 years. In 32/35 patients with OPLL and 23/25 patients with CSM, myelopathy improved. Improvements in JOA scores were maintained at last follow up in 26/35 patients with OPLL and 21/25 patients with CSM. Late neurologic deterioration occurred in 10 patients with OPLL at a mean of eight years after surgery and in four patients with CSM (including 3 patients with athetoid cerebral palsy) at a mean of 11 years postop. Overall, short-term results of laminoplasty were maintained at 10 years and Seichi's group concluded that double-door laminoplasty is a reliable procedure for patients with cervical myelopathy (except in those with athetoid cerebral palsy).

Neurologic recovery is most likely related more to preoperative neurologic status and degree of myelopathy than the specific laminoplasty technique performed. No significant difference has been demonstrated with one laminoplasty technique compared to the other. The etiology of stenosis, however, does appear to have an effect on prognosis following laminoplasty. The benefits of surgery in patients with CSM appear to last in the long term, but there is a slightly higher rate of late clinical deterioration in patients with OPLL. Age greater than 60 and a history of symptoms preoperatively for more than one year are also poor prognostic indicators [42, 78].

 There are very few studies directly comparing surgical options for cervical myelopathy. Kaminsky's group compared outcomes in patients treated with laminoplasty versus laminectomy without fusion for CSM using the modified Nurick grading scale [93]. Both groups improved to a similar degree. The patients who underwent laminoplasty, however, had less postoperative cervical pain and less cervical range of motion. Other earlier studies also found no difference in long-term neurologic outcomes between patients treated with laminectomy without fusion and laminoplasty [12, 56, 63]. However, Heller et al. performed a retrospective review of two matched groups of patients with multilevel cervical myelopathy who underwent either laminectomy with fusion or laminoplasty [94]. Compared to the laminectomy and fusion cohort, the laminoplasty cohort showed greater rates of objective improvement in function as judged by Nurick scores and greater subjective improvement in strength, dexterity, sensation, pain, and gait. In addition, no complications were noted in the laminoplasty cohort compared to 14 complications in nine patients who underwent laminectomy and fusion. Heller et al. concluded that the differences he found in terms of complications and functional improvement between the two cohorts suggest that laminoplasty may be more effective and safer than laminectomy with fusion for multilevel cervical myelopathy.

Edwards et al. compared laminoplasty and anterior decompression and fusion in a matched cohort of 13 patients in each group and found higher rates of neurologic improvement, less pain medication needs, and fewer complications in the laminoplasty cohort [95]. They concluded that though both multilevel corpectomy and laminoplasty effectively arrest progression of myelopathy and lead to neurologic improvement, laminoplasty is a better option. Wada's group also retrospectively compared long-term outcomes between 23 patients treated with subtotal corpectomy and 24 patients treated with laminoplasty for multilevel CSM over 10–14 years [48]. Neurologic recovery was identical between the two groups and was usually maintained for more than ten years. However, the subtotal corpectomy group had longer surgeries, more blood loss, and a 26% pseudarthrosis rate. Axial pain was significantly more common in the laminoplasty group compared to the corpectomy group, at 40% and 15%, respectively. Loss of cervical motion was more severe in the laminoplasty group as well: range of motion was 29% of what it was preoperatively in this group, compared to 49% in the corpectomy group. Finally, Yonenobu et al. also compared the results of 41 patients with CSM undergoing either subtotal corpectomy with strut grafting and 42 patients who underwent laminoplasty with a minimum followup of 2 years [44]. There was no significant difference in recovery rate and final score in terms of JOA scores. Complications were more frequent in the subtotal corpectomy group, however, and these were usually due to bone-graft-related issues. Yonenobu's group concluded that in terms of neurologic results, complications, and the potential for immediate mobilization that laminoplasty affords, it is the preferred surgical technique for patients with CSM.

## 18. Conclusion

Laminoplasty is becoming an increasingly popular treatment for multilevel cervical stenosis due to cervical spondylotic myelopathy, OPLL, and other causes. Laminoplasty minimizes the risk of certain complications associated with other surgical options, such as graft and fusion-related complications, postoperative kyphosis and instability, and the morbidity of an anterior approach. Laminoplasty does have its own set of potential complications, including laminar closure, axial neck pain, nerve root palsies, and loss of cervical motion and alignment. However, laminoplasty techniques are continuously being refined to address such potential shortcomings. Indeed, further prospective data with longer-term followup comparing laminoplasty techniques to other surgical options is necessary. Yet, outcomes in laminoplasty patients that are at least as good as anterior decompression and fusion and laminectomy can be expected. In the appropriate patient and with proper surgical technique, laminoplasty can be an excellent option for patients with multilevel cervical stenosis and myeloradiculopathy.
